# The transcriptomes of novel marmoset monkey embryonic stem cell lines reflect distinct genomic features

**DOI:** 10.1038/srep29122

**Published:** 2016-07-07

**Authors:** Katharina Debowski, Charis Drummer, Jana Lentes, Maren Cors, Ralf Dressel, Thomas Lingner, Gabriela Salinas-Riester, Sigrid Fuchs, Erika Sasaki, Rüdiger Behr

**Affiliations:** 1 Platform Degenerative Diseases, German Primate Center – Leibniz Institute for Primate Research, Kellnerweg 4, 37077 Göttingen, Germany; 2 Institute of Cellular and Molecular Immunology, University Medical Center Göttingen (UMG), Humboldtallee 34, 37073 Göttingen, Germany; 3 DZHK (German Center for Cardiovascular Research), Partner Site Göttingen, Germany; 4 Microarray and Deep-Sequencing Core Facility, University Medical Center Göttingen (UMG), Justus-von-Liebig-Weg 11, 37077 Göttingen, Germany; 5 Department of Human Genetics, University Medical Center Hamburg-Eppendorf, Martinistraße 52, 20246 Hamburg, Germany; 6 Department of Applied Developmental Biology, Central Institute for Experimental Animals, 3-25-12 Tonomachi Kawasaki-ku, Kawasaki, 210-0821 Japan; 7 Keio Advanced Research Center, Keio University, Shinjuku-ku, Tokyo, Japan

## Abstract

Embryonic stem cells (ESCs) are useful for the study of embryonic development. However, since research on naturally conceived human embryos is limited, non-human primate (NHP) embryos and NHP ESCs represent an excellent alternative to the corresponding human entities. Though, ESC lines derived from naturally conceived NHP embryos are still very rare. Here, we report the generation and characterization of four novel ESC lines derived from natural preimplantation embryos of the common marmoset monkey (*Callithrix jacchus*). For the first time we document derivation of NHP ESCs derived from morula stages. We show that quantitative chromosome-wise transcriptome analyses precisely reflect trisomies present in both morula-derived ESC lines. We also demonstrate that the female ESC lines exhibit different states of X-inactivation which is impressively reflected by the abundance of the lncRNA *X inactive-specific transcript* (*XIST*). The novel marmoset ESC lines will promote basic primate embryo and ESC studies as well as preclinical testing of ESC-based regenerative approaches in NHP.

Embryonic stem (ES) cells are generally derived from the inner cell mass of preimplantation embryos at the blastocyst stage^1^,^2^,^3^,^4^,^5^,^6^,^7^. If cultured under appropriate conditions, ES cells can proliferate indefinitely while maintaining a pluripotent state^1^. They can give rise to all somatic cell types deriving from the embryonic germ layers, i.e. ectoderm, mesoderm and endoderm, as well as germ cells.

Due to their *in vitro* differentiation capability human ES cells hold great promise for curative cell replacement approaches^8^, reviewed in refs 9, 10, 11, 12. However, before pluripotent stem cell-based therapies may become a routine clinical option to treat specific diseases, biosafety and efficacy issues have to be tested thoroughly. Due to the close phylogenetic relationship of non-human primates (NHP) to humans, NHP ES cells represent an excellent alternative to human ES cells with regard to basic and preclinical ES cell research reviewed in refs 13,14 and allow experiments not possible with human embryos^15^. Moreover, studies in NHP are often more relevant with regard to the human than studies in more distant species, which do not always reflect human physiology and anatomy in an adequate way reviewed in refs 16, 17, 18, 19. Preclinical testing of ES cell-based regenerative medicine would benefit from appropriate NHP models.

Rhesus (*Macaca mulatta*) and cynomolgus (*Macaca fascicularis*) macaques belong to the group of Old World monkeys and are the most frequently used NHP species in biomedical research^20^. The first non-human primate embryonic stem cell line was derived from rhesus macaque in 1995^3^.

The common marmoset monkey (*Callithrix jacchus*) is a New World monkey endemic to Brazil. It has several significant practical and biological advantages compared to macaques. The marmoset monkey is small, easy to handle and free of zoonoses. These characteristics contribute to relatively low housing costs. Additionally, common marmosets have a significantly shorter generation time as well as a higher fecundity than macaques. Therefore, the common marmoset is the NHP model of choice for specific applications and purposes and nicely complements the macaque models. This is reflected by many studies performed in the fields of stem cell research^21^,^22^, reproductive biology^23^,^24^,^25^, developmental biology^26^,^27^, and neurobiology reviewed in refs 28,29. Embryo-derived pluripotent stem cells of the common marmoset were first published in 1996^2^. After this initial publication of eight cell lines, the generation of three additional marmoset monkey ES cell lines was reported in 2005^6^. The currently published set of marmoset ES cell lines was completed in 2009 when we published an additional marmoset ES cell line which was originally also generated by Erika Sasaki and colleagues^7^. Unfortunately, all marmoset ES cell lines generated by Thomson *et al*.^2^ are not available anymore (E-mail communication with WiCell, which served as distributor of these cells).

Here, we report the generation and characterization of four novel marmoset monkey ES cell lines derived from natural preimplantation embryos. Importantly, three of the ES cell lines were derived from morula stages, which has not been reported so far. We characterized the novel ES cell lines in comparison with the established marmoset ES cell line cjes001^7^.

## Materials and Methods

### Animals and embryo collection

Marmoset monkeys (*Callithrix jacchus*) were obtained from the self-sustaining breeding colony of the German Primate Center (Deutsches Primatenzentrum; DPZ) and kept as described previously^30^. All aspects of this study were approved by an external ethics committee (Niedersächsisches Landesamt für Verbraucherschutz und Lebensmittelsicherheit, license numbers AZ 42502-04/066/06 and AZ 42502-04-14/1462). All methods were carried out in accordance with the approved guidelines.

Female common marmosets (n = 3) in the age range of 3.9 to 7.3 years were used as embryo donors. All females were kept pairwise with fertile males, and their reproductive cycles were basically monitored as described previously^31^. Collection of natural embryos was performed by flushing the uterus on day 4 to 6 after ovulation to obtain preimplantation embryos. The uterus was flushed either by invasive or minimal-invasive methods^31^. After invasive surgery the animals received antibiotic therapy (Duphamox 0.1 ml i.m.), and analgesic medication was given 30 minutes before surgery (Metacam 0.1 ml s.c.). The awakening period under a warming infrared lamp was monitored carefully and animals were retransferred to their cages only after full recovery. Invasive surgery was applied maximally six times per animal, and only to those animals with at least four unsuccessful previous minimal-invasive embryo collection attempts. Minimal-invasive embryo collection was performed under short term anesthesia with Diazepam (0.05 ml/animal) and Alfaxan (0.1 ml/100 g bodyweight).

### Cell Culture

#### Mouse embryonic fibroblasts (MEFs)

Gamma-irradiated MEFs served as feeder cells and were obtained as described previously^30^.

#### Initial culture of recovered embryos

After recovery, preimplantation embryos were transferred to and kept in embryonic stem cell medium (ESM)^30^ [KO-DMEM (Gibco), 20% (v/v) KnockOut Serum Replacement (Gibco), 1% (v/v) Penicilline/Streptomycine (Gibco), 0,1% (v/v) Amphotericin B (Gibco), 1% (v/v) MEM Non-Essential Amino Acids Solution (Gibco), 2 mM GlutaMAX (Gibco), 50 μM 2-mercaptoethanol (Gibco)] until removal of the zona pellucida (ZP). All *ex vivo* processing of the embryos was done at 37 °C. The ZP was removed using pronase (2 mg/mL, Sigma #P8811) dissolved in KO-DMEM (Gibco). Embryos were first washed in a 100 μL drop of pronase solution, then transferred into another drop of pronase solution and kept there for 1–3 min until degradation of the ZP was observed. ZP-free embryos were immediately washed sequentially in four drops of ESM to remove the pronase and finally transferred onto MEFs in a 35 mm diameter well with ESM. Embryos were allowed to attach without any disturbances for three days before cultures were checked. If primary outgrowths were observed, the culture was continued for 2 to 3 weeks until further passaging. All pluripotent cells were cultured under hypoxic conditions (37 °C, 8% CO_2_, 5% O_2_) in ESM, and medium was changed every two to three days. Passaging of primary outgrowths and of resulting ES cells is described below.

#### Expansion and maintenance of embryonic stem cells

For further passaging of the primary outgrowths and ES cells, StemPro Accutase (Life Technologies, #A11105-01) was used. Briefly, cells of one well in a six-well plate were washed with PBS and incubated with 1 mL Accutase at 37 °C for 4 min. The cell suspension was transferred to 5 mL of pre-warmed ESM and the remaining feeder layer was washed with 3 mL ESM. Cells were pelleted (5 min, 200 × g, RT), resuspended in ESM and seeded onto fresh MEFs. Medium was changed every two to three days.

#### PCR for the detection of pluripotency associated genes

Oligonucleotides (Sigma) used for detection of mRNA coding for pluripotency associated genes are listed in Table S1. KOD Hot Start DNA Polymerase from Novagen was used according to manufacturer’s instructions.

#### Immunofluorescence

Immunofluorescence stainings were performed as described previously^30^. Antibodies and their dilutions are listed in Table S2.

#### AP live stain

For detection of Alkaline Phosphatase (AP), Alkaline Phosphatase Live Stain (Life Technologies, #A14353) was used. Briefly, growth medium was removed and the culture was washed with pre-warmed DMEM/F-12 two times for 2–3 minutes. Then a 1X AP Live Stain working solution was applied directly on to the cell culture and incubated for 20–30 minutes. The AP Live Stain was removed and pre-warmed DMEM/F-12 was applied to the culture prior to the visualization of fluorescent-labeled colonies under fluorescent microscopy using a standard FITC filter. Images were captured immediately.

#### Teratoma formation and analysis

For teratoma formation, 1–2 × 10^5^ mouse embryonic feeder cells were combined with 8–9 × 10^5^ ES cells in a final volume of 70 μL PBS. 60–75 μL Matrigel (Corning, #354277) were added to this cell suspension and injected subcutaneously into the inguinal region of male immunodeficient SCID/beige mice. Teratomas were retrieved 10–17 weeks, in one case 24 weeks after injection. Teratomas were immediately fixed after recovery in Bouin’s solution. After paraffin embedding, they were sectioned at 5 μm. Sections were then Hematoxylin and Eosin stained or processed for immunohistochemistry as described previously^30^.

#### Karyotyping

Karyotyping was performed by the Cytogenetic Laboratory in the Department of Human Genetics at the Universitätsklinikum Hamburg-Eppendorf (Germany) according to standard procedures. Briefly, for each cell line chromosome preparation was done from two or three 35 mm wells with ES colonies. ES cells from the wells were pooled before analysis. Then the cells were arrested with 0.2 μg/ml colcemid for 3 h and dissociated with 0.25% trypsin EDTA. For hypotonic treatment, cells were subjected to 55 mM KCl and fixed with methanol/acetic acid (3:1). For each cell line, 15 metaphases from GTG banded chromosome spreads were analysed under a light microscope at a 1000× magnification and at least four metaphases were karyotyped using a cytogenetic image analysis system (CytoVysion; Leica Biosystems). Karyotyping was done according to the chromosome assigning of Neusser *et al*.^32^. In case of suspected mosaicism or clonal aberration, 30 metaphases were analysed.

#### Transcriptome analysis

Two independent biological replicates were analyzed for each cell line. For sequencing, the RNA-samples were prepared with the “TruSeq RNA Sample Prep Kit v2” according to the manufacturer’s protocol (Illumina). Single read (50 bp) sequencing was conducted using a HiSeq 2000 (Illumina). Sequences were aligned to the genome reference sequence of *Callithrix jacchus* (Ensembl genome assembly 3.2.1) using the STAR alignment software (version 2.3.0e)^33^ allowing for 2 mismatches within 50 bases. Subsequently, filtering of unique hits and counting was conducted with SAMtools (version 0.1.18)^34^ and HTSeq (version 0.6.1p1)^35^. Read counts were analyzed in the R/Bioconductor environment (version 3.2, www.bioconductor.org) using the DESeq2 package (version 1.8.1)^36^. Candidate genes were filtered to a minimum of 2-fold change and FDR-corrected p-value < 0.05. Gene annotation was performed using *Callithrix jacchus* entries from Ensembl v78 (www.ensembl.org) via the biomaRt package (version 2.24.0)^37^. GO enrichment analysis on candidate genes was conducted with the Goseq package (version 1.2)^38^ using standard parameters. The data discussed in this paper are generated in compliance with the MIAME guidelines and have been deposited in NCBI’s Gene Expression Omnibus, accessible through GEO Series accession number GSE70897.

## Results

### Establishment and culture of novel embryonic stem cell lines

**Figure 1:**
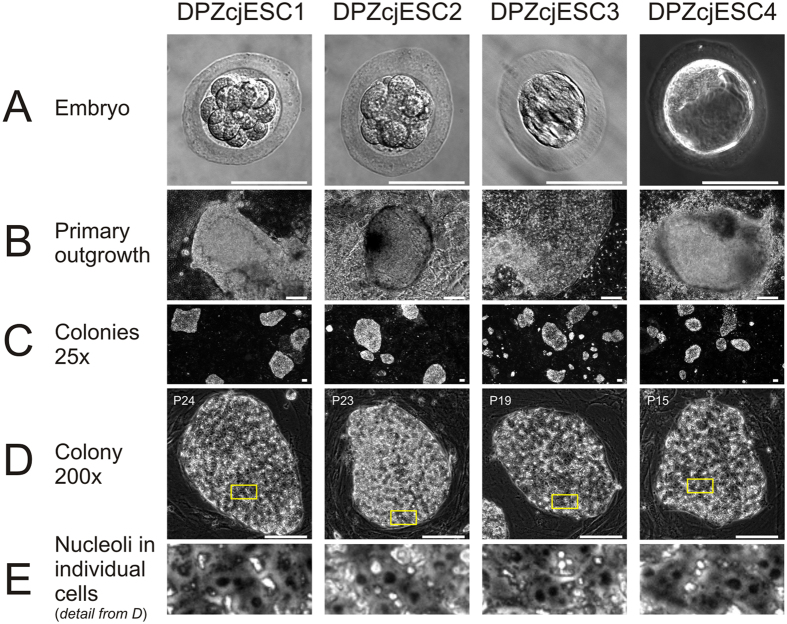
Derivation and morphology of the established ES cell lines. (**A**) Embryos were obtained from adult common marmoset monkeys 4–6 days after ovulation. (**B**) Primary outgrowths on mouse embryonic feeder cells 13–15 days after plating of the embryo. (**C**) Colony overview, magnification 25-fold. (**D**) Single ES cell colony, magnification 200-fold. Individual passage number (P) is indicated in the upper left. (**E**) Magnification of framed area in D showing the prominent nucleoli. All bars = 100 µm.

We established four embryonic stem cell lines, designated DPZcjESC1-4. The embryos were obtained from different donors (Table 1). Three of our novel lines were derived from morulae. DPZcjESC1 and 2 were obtained from early, non-compacted morulae, while DPZcjESC3 was obtained from a compacted morula. One line (DPZcjESC4) was derived from an expanded blastocyst stage embryo (Fig. 1A). In case of blastocysts, the embryos were plated onto the MEF layer with intact trophoblast. Immunosurgery to remove the trophoblast was not performed. All embryos formed clearly identifiable primary outgrowths (Fig. 1B). After passaging of the primary outgrowths and expansion on mouse embryonic feeder cells, all lines stably proliferated and exhibited the typical morphology of common marmoset pluripotent stem cells^2^,^6^,^7^,^30^,^39^. The cell lines formed tightly packed, flat colonies (Fig. 1C,D). Individual cells can be distinguished within a colony and the ES cells exhibit a high ratio of nucleus to cytoplasm. Prominent nucleoli are clearly visible (Fig. 1E). All cell lines tolerate enzymatic colony dissociation during passaging, which is done every six to 13 days. All cell lines tolerated cryopreservation, thawing and subsequent re-culturing.

### Expression of pluripotency markers

**Figure 2:**
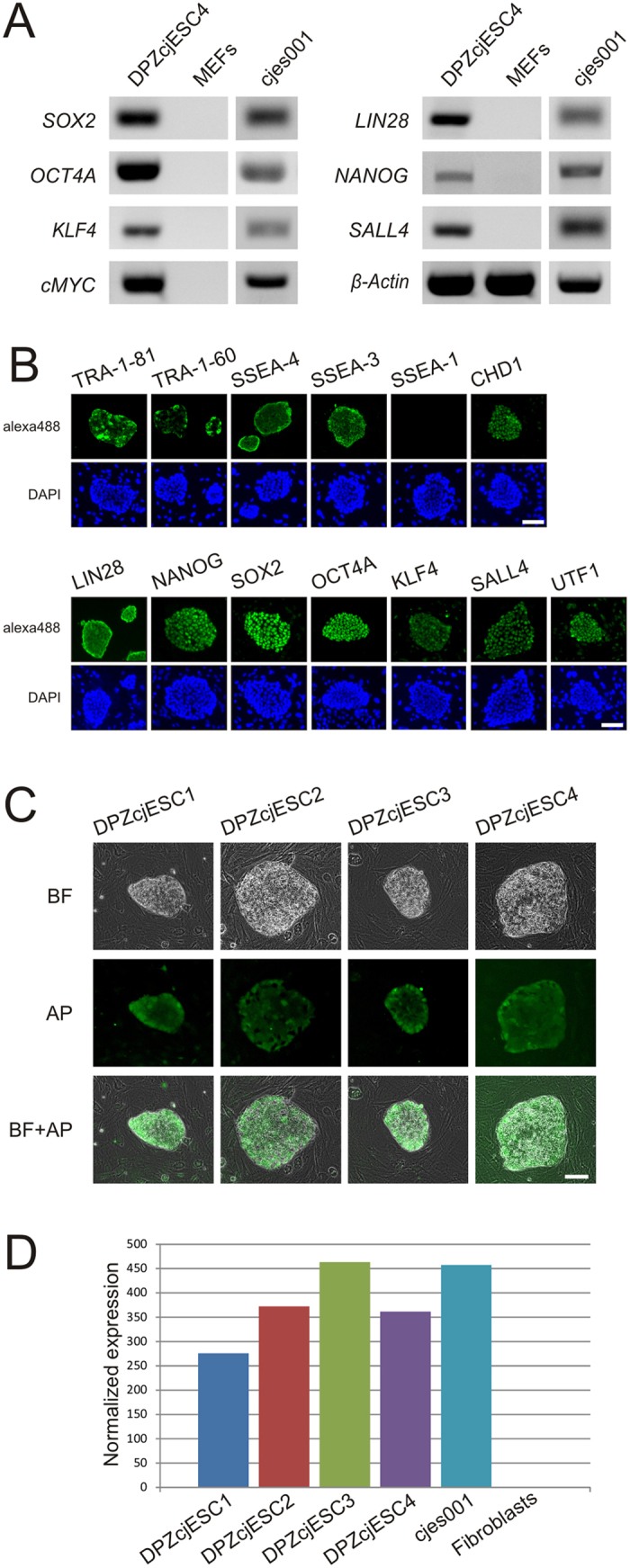
Pluripotency marker expression. (**A**) PCR analysis of the generated ESC line DPZcjESC4. The ESC line expressed mRNA coding for well-established pluripotency-associated factors. Mouse embryonic feeder cells (MEFs) were used as negative control and the established embryonic stem cell line cjes001^7^ as positive control. (**B**) Immunofluorescence staining of ES cell colonies. Antibodies directed against pluripotency-associated epitopes confirmed expression of the surface molecules TRA-1-81, TRA-1-60, SSEA-4 and SSEA-3, the Chromodomain Helicase DNA Binding Protein 1 (CHD1), the RNA binding protein LIN28 and several transcription factors (NANOG, SOX2, OCT4, KLF4, SALL4, UTF1). As expected, SSEA-1 was not detected. (**C**) Alkaline Phosphatase (AP) live staining. Expression of AP was detected in all cell lines. (**D**) Normalized Telomerase expression (as obtained from the DeSEQ software) in the four novel ES cell lines DPZcjESC1-4, the established ES cell line cjes001 and primary skin fibroblasts. Transcripts coding for the Telomerase enzyme were detected only in ES cells. Data was generated by deep sequencing and analyzed with the DESeq2 package^36^. All bars = 100 µm.

To demonstrate the expression of characteristic pluripotency markers by the cell lines, we performed RT-PCR and immunofluorescence. On the mRNA level, we detected expression of *SOX2*, *OCT4*, *KLF4*, *cMYC, NANOG,* all being transcription factors, and *LIN28,* an RNA binding protein. All factors have been used for reprogramming of marmoset monkey somatic cells to a pluripotent state^30^,^39^. Messenger RNA coding for the pluripotency-associated transcription factor *SALL4* was also detected. (Fig. 2A, Fig. S3). Expression of most of these marker genes was confirmed on the protein level by immunofluorescence (Fig. 2B). The ES cell lines also express the cell surface antigens TRA-1-81, TRA-1-60, SSEA-4 and SSEA-3 (Fig. 2B, Fig. S4) which identify pluripotent cells^40^. Moreover, expression of the transcription factor UTF1^41^,^42^ as well as the Chromodomain Helicase DNA Binding Protein 1 (CHD1)^43^,^44^ was detected. Expression of the cell surface antigen SSEA-1, characteristic of mouse, but not primate pluripotent cells, was not detected (Fig. 2B, Fig. S4). All ES cell lines expressed alkaline phosphatase (Fig. 2C) and telomerase (Fig. 2D).

### Differentiation assay demonstrates pluripotency

**Figure 3:**
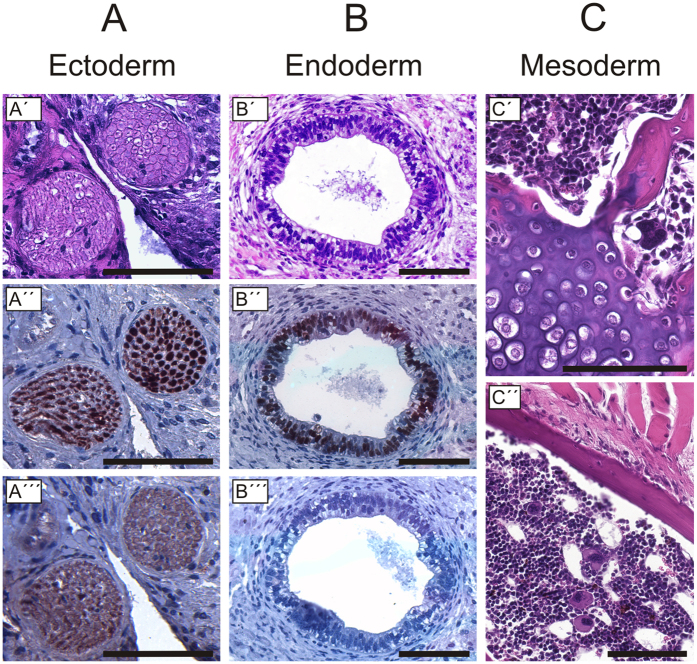
Teratoma assay to test for pluripotency. HE staining and immunohistochemical analysis of teratoma tissue derived from the cell line DPZcjESC4. HE staining demonstrates the presence of nerves (**A**), endodermal epithelium (**B**) and of mesodermal derivatives (**C**). (**A**′) Cross sections of peripheral nerves. (**A**′′) Detection of the neuronal marker β-Tub III. (**A**′′′) Isotype control for (**A**′′). (**B**′) Cross sections of a cyst lined with endodermal gut-like epithelium. (**B**′′) Detection of SOX9, which is a marker of endodermal progenitor cells. (**B**′′′) Isotype control for (**B**′′). (**C**′) Cartilage (lower left area) and bone trabecula (upper right corner). (**C**′′) Bone trabecula (upper part) and bone marrow including megakaroycytes (lower part). All bars = 100 µm.

To investigate the *in vivo* differentiation potential, we injected the ES cells into immunodeficient SCID/beige mice. Fig. 3 shows histological analysis of tumor tissue developed from the cell line DPZcjESC4. Ectodermal differentiation was indicated by neural tissue (Fig. 3A). Endoderm was represented by primitive gut-like epithelium (Fig. 3B) and mesoderm was represented by osteogenic (Fig. 3C′,C″), chondrogenic (Fig. 3C′) and hematopoietic differentiation (Fig. 3C″). Ectodermal differentiation was confirmed by immunohistochemical detection of β-Tubulin 3 (β-TUB III, ectoderm; Fig. 3A″) and endodermal differentiation by detection of SOX9 (Fig. 3B″, which is a marker of endodermal stem and progenitor cells). Histological analyses of teratoma tissue developed from the cell lines DPZcjESC1-3 are shown in Supplemental Figure S5. Together, these data show the pluripotent differentiation potential of the ES cells.

### Gene expression levels in morula- and blastocyst-derived cell lines

**Figure 4:**
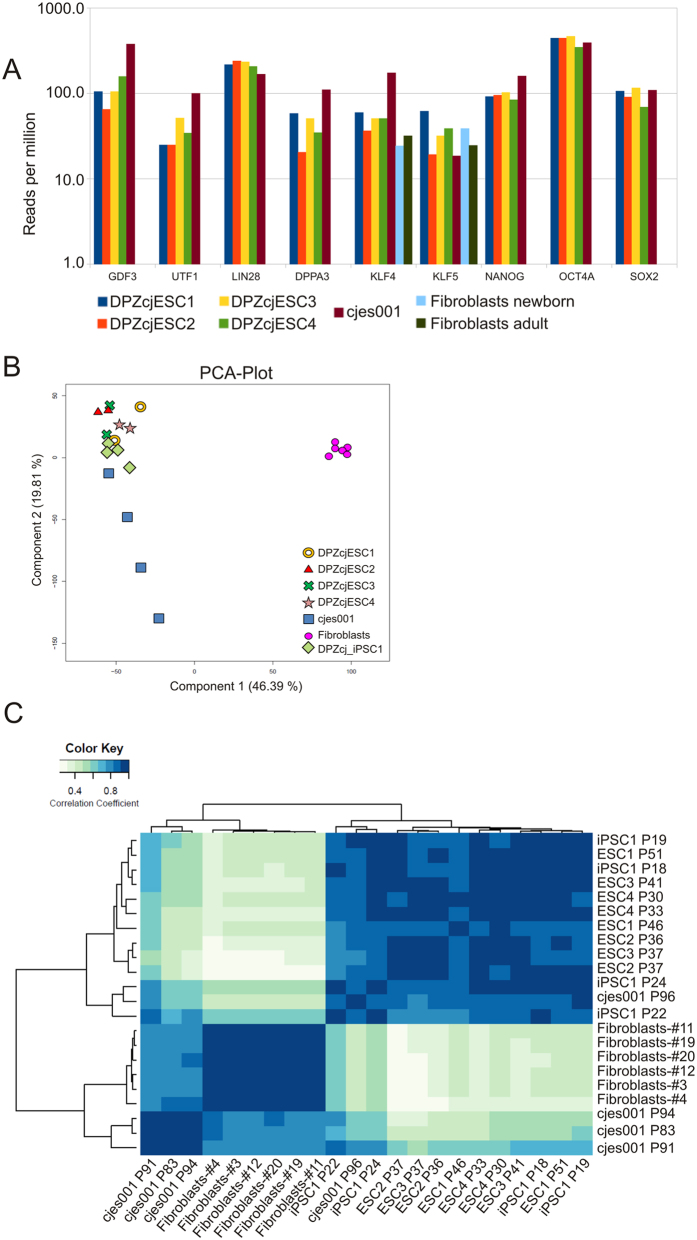
Transcriptome analysis. (**A**) Expression levels of selected pluripotency markers. Levels of mRNA for *OCT4*, *SOX2*, *NANOG*, *LIN28*, *KLF4*, *KLF5*, *UTF1*, *DPPA3 (Stella*), and *GDF3* in all four generated ES cell lines (DPZcjESC1-4) and in marmoset skin fibroblasts were compared to the levels in the established embryonic stem cell line cjes001. (**B**) Principal Component Analysis (PCA) plot of the four generated ES cell lines DPZcjESC1-4, the established ES cell line cjes001, the marmoset iPS cell line DPZcj_iPSC1, and primary skin fibroblasts showing the clustering of samples in gene expression space. Numbers in parentheses represent the percentage of total data variance covered by a principal component. (**C**) Heat map and hierarchical clustering of the normalized transcriptome expression profile for the generated ES cell lines DPZcjESC1-4, the previously generated iPS cell line DPZcj_iPSC1, the established ES cell line cjes001 and marmoset monkey fibroblasts. Darker color (blue) indicates higher correlation of gene expression.

There were five ES cell lines (four novel and the reference cell line published previously^7^) available for this study. Two cell lines were derived from blastocysts (DPZcjESC4 and cjes001) and three from morula stages (DPZcjESC1-3). We performed whole transcriptome analyses of the marmoset ES cells by deep sequencing and included primary marmoset monkey fibroblasts as control. We were interested in whether the embryonic origin of the ES cell lines was reflected by differential mRNA expression of selected pluripotency markers by the corresponding ES cell lines. Hence, we analyzed expression levels of *OCT4*, *SOX2* and *NANOG* as core pluripotency factors and further included *LIN28*, *KLF4*, *KLF*5, *UTF1*, *DPPA3* (*Stella*), and *GDF3*. Analyses of the transcriptome data revealed similar relative expression levels of all markers analyzed in the individual samples of both the morula and the blastocyst-derived cell lines (Fig. 4A). To investigate whether global differences exist between the cell lines of different embryonic origin we performed a principle component analysis (PCA) providing a general overview (Fig. 4B): All four novel ES cell lines closely clustered together, irrespective of their embryonic origin. While the cell transcriptomes of different passages of the established ES cell line cjes001 had a higher data variance regarding component 2, the scatter was very low regarding the more indicative component 1 (46.39% variance). However, all ES cell lines were clearly separated from the fibroblasts. Hierarchical clustering on expression profiles confirmed that the novel ES cell lines are very similar but that their transcriptomes differ from the transcriptomes of the established ES cell line cjes001 (Fig. 4C). In a previous study where we generated common marmoset iPS cells by non-viral means^30^, hierarchical clustering showed that our iPS cells clustered apart from the cjes001. Interestingly, combining the global transcriptome data of both studies (the present and^30^) revealed that both our ES cells and iPS cell line clustered together and are more distant from the fibroblasts than cjes001 is (Fig. 4C).

### Karyotyping

To the best of our knowledge, there is no official agreement on the chromosome assignment and nomenclature for the common marmoset monkey being comparable to the International System for Human Cytogenetic Nomenclature (ISCN, latest version 2013) which is updated every five years^45^. There is one study by Sherlock *et al*. published in 1996 suggesting a nomenclature^46^. However, it is very difficult to distinguish the g-banded chromosomes in the corresponding publication making an unambiguous chromosome assignment problematic. Therefore we refer to the study by Neusser *et al*.^32^ which in turn is relying on the nomenclature proposed by Sherlock *et al*.^46^. However, it must be pointed out that we are not able to unambiguously bring in line the banded chromosomes 19 and 20 from Neusser *et al*.^32^ with the idiograms presented in Sherlock *et al*.^46^.

**Figure 5:**
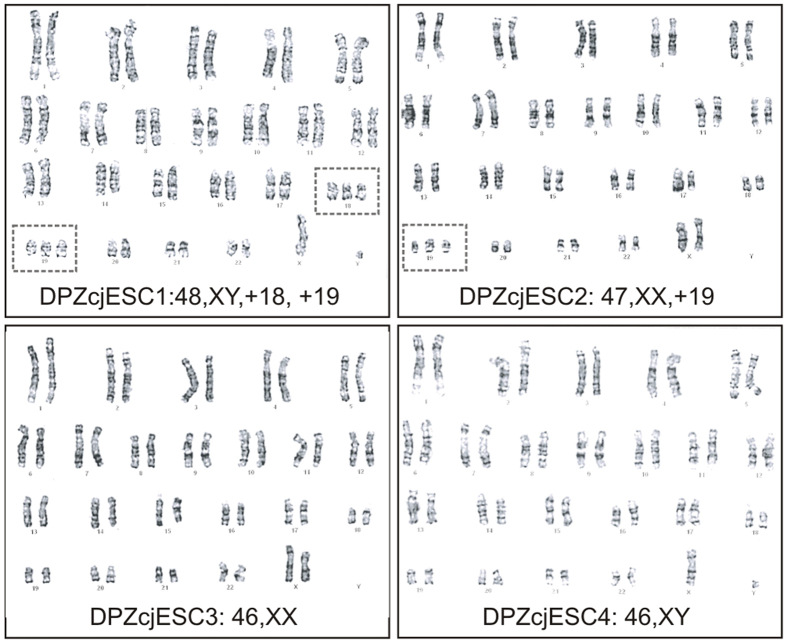
Karyotyping and determination of the sex. Karyotype analysis of the four generated lines DPZcjESC1-4. The male cell line DPZcjESC1 displayed a trisomy for chromosomes 18 and 19 (48, XY, +18, +19), the female cell line DPZcjESC2 displayed a trisomy for chromosome 19 (47, XX, +19) and the cell lines DPZcjESC3 (female) and DPZcjESC4 (male) showed a normal karyotype (46, XX and 46, XY). Trisomic chromosomes are boxed.

Irrespective of the ambiguity associated with the assignment of marmoset chromosomes 19 and 20, we subjected all four ES cell lines to karyotype analysis. While the cell lines DPZcjESC3 and DPZcjESC4 had a normal female (46,XX) and male (46,XY) karyotype respectively, DPZcjESC1 (male) displayed a trisomy for chromosomes 18 and 19 (48,XY, +18, +19) and DPZcjESC2 (female) a trisomy for chromosome 19 (47,XX, +19) (Fig. 5).

### The transcriptomes of the generated ES cells reflect their genomic status

**Figure 6:**
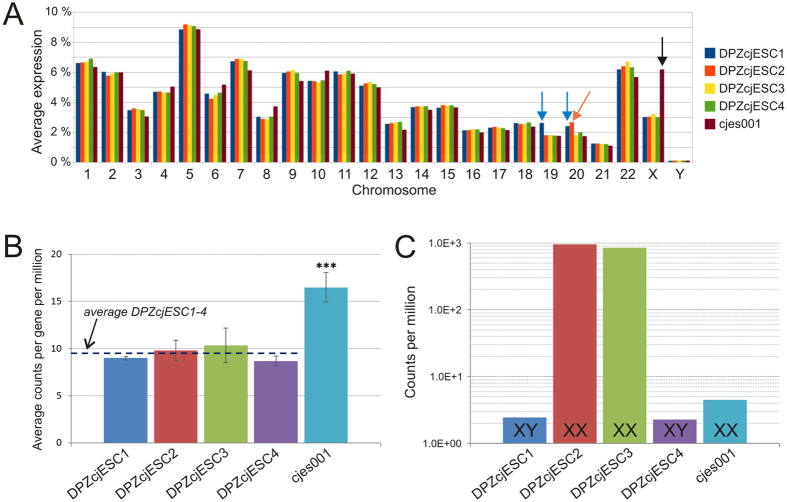
Global transcriptome analysis. (**A**) Relative average expression per chromosome. The relative average expression per chromosome was calculated from the sum of all reads from one particular chromosome divided by the sum of all reads from all chromosomes for each of the five ES cell lines. Blue arrows indicate significantly elevated expression from chromosomes 19 and 20 for DPZcjESC1, the orange arrow indicates significantly elevated expression from chromosome 20 for DPZcjESC2 and the black arrow indicates elevated expression from the X chromosome for cjes001. (**B**) Average expression of X-linked genes. Average reads per gene located on the X chromosome per million reads in the samples from the generated ES cell lines DPZcjESC1-4 and the established ES cell line cjes001. With 16.5 reads per million for the established female cell line cjes001 the average expression was 1.7-fold as high as for the four generated ES cell lines with an average of 9.5 reads per million (p-value of the t-tests was 3.29*10^−6^). C) *X-inactive specific transcript* (*XIST*)-expression. Number of reads mapping to the *XIST*-coding region detected in the samples from the generated ES cell lines DPZcjESC1-4 and the established ES cell line cjes001. Less than 3 reads per million were detected in the two male ES cell lines DPZcjESC1 and 4 whereas both female lines (DPZcjESC2 and 3) expressed *XIST* robustly (945 and 851 reads per million). In contrast, in the established ES cell line cjes001 *XIST* was not abundant (less than 5 reads per million).

Irrespective of their karyotype, all generated ES cell lines were indistinguishable from each other in all assays performed so far. Therefore we were wondering whether the additional copies of two chromosomes in DPZcjESC1 and of one chromosome in DPZcjESC2 might be subjected to regulatory mechanisms which lead to at least partial inactivation of the respective chromosome. This phenomenon of dosage compensation is known for the X chromosome (see below). Therefore we compared the relative expression per chromosome (calculated from the sum of all reads from one particular chromosome divided by the sum of all reads from all chromosomes) between the five ES cell lines. We found that the additional chromosomes are not subject to chromosome-wide regulatory mechanisms in the sense of dosage compensation since trisomies are clearly reflected by the deep sequencing data. Indeed, the relative expression of DPZcjESC1 was approximately 50% higher for two chromosomes, identified as 19 and 20 (p ~8 × 10^−12^ (paired t-test), blue arrows in Fig. 6A) in the transcriptome analysis. Likewise, the relative expression of DPZcjESC2 was ~1.5-fold (p = 1.26 × 10^−6^, (paired t-test), orange arrow in Fig. 6A) for chromosome 20. No such difference was found for any other chromosome that was present in disomy. Remarkably, karyotype analysis revealed trisomies for chromosomes 18 and 19 in DPZcjESC1 (instead of 19 and 20 as transcriptome analysis did) and a trisomy for chromosome 19 in DPZcjESC2 (instead of 20 as transcriptome analysis did).

We conclude that (i) two of our four new ES cell lines show trisomies, (ii) there is no evident compensatory gene expression (down-) regulation due to the presence of an additional copy of a chromosome, (iii) the transcriptome of the ES cell lines reflects their karyotype, (iv) it needs to be determined which chromosomes are affected (by clarification of numbering of the marmoset monkey chromosomes).

### Different conditions of X-inactivation in female ES cell lines

Interestingly, comparison of the relative expression per chromosome between the five ES cell lines did not only confirm the trisomies determined by karyotyping. It also revealed that the average expression from the X-chromosome in the established female cell line cjes001 was much higher than in the novel ES cell lines (Fig. 6A, black arrow). That prompted us to analyze this issue in more detail. Quantitative analysis of the transcriptome data showed that the average expression of each gene on the X-chromosome in the established female cell line cjes001 was increased 1.7-fold compared to the average expression in the four novel ES cell lines (16.5 reads per million for cjes001 vs. an average of 9.5 reads per million for DPZcjESC1-4, p = 3.29 × 10^−6^, paired t-test) (Fig. 6B). *X-inactive specific transcript* (*XIST*) is a large long non-coding RNA known to be an important effector in the process of X inactivation^47^. We were wondering whether the significantly different average expression of the genes on the X chromosome would be associated with differences in the expression levels of *XIST*. Indeed, analysis of the transcriptome data revealed an evident correlation between *XIST*-expression and transcription activity from the X chromosome. While the relative amount of detected transcripts from the X chromosome was comparable in all ES cell lines generated in this study (Fig. 6A for an overview and Fig. 6B in higher resolution), almost no *XIST* (<3 reads per million) was detected in the two male ES cell lines (DPZcjESC1 and 4) whereas both female lines (DPZcjESC2 and 3) expressed *XIST* at high levels (945 and 851 reads per million) (Fig. 6C). In sharp contrast, in the previously established female ES cell line cjes001 where quantitative analysis of the transcriptome data indicated a significantly elevated transcriptional activity from the X chromosome, *XIST* was also almost absent being in the same range as in the male ES cell lines (<5 reads per million) (Fig. 6C).

We conclude that X-inactivation can occur in female ES cells of the common marmoset monkey and that X-inactivation correlates with expression of *XIST.* However, X-inactivation seems not to be an essential inherent characteristic of female ES cells.

### Common marmoset ES cells exhibit features of primed pluripotency

**Figure 7:**
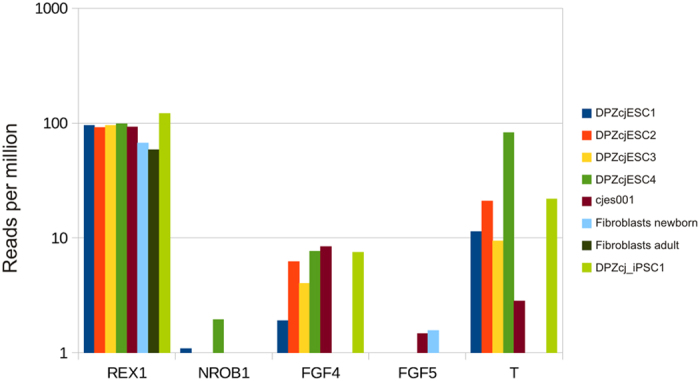
Average expression of genes indicative of naïve or primed pluripotency. Levels of mRNA for *REX1*, *NROB1*, *FGF4*, as markers of naïve pluripotency and *FGF5*, and Brachyury (T) as genes associated with primed pluripotency in all four generated ES cell lines (DPZcjESC1-4), the previously generated iPS cell line DPZcj_iPSC1 and in marmoset skin fibroblasts were compared to the levels in the established embryonic stem cell line cjes001.

Our novel ESC lines exhibit morphological characteristics of primed pluripotency, as the colonies are flat (Fig. 1) and not dome-shaped^48^. Further substantiating a primed state, the cells lack SSEA-1 expression and have an inactivated X chromosome^48^. In order to obtain insights into the molecular state of pluripotency (naïve versus primed) of the novel ES cell lines, we analyzed the presence of selected marker transcripts including *REX1* (*ZFP42*), *NROB1*, *FGF4*, *FGF5*, and *T* (*Brachyury*) (Fig. 7). Since the regulation of pluripotency in the mouse is distinct from the regulation in primates^27^,^48^,^49^,^50^, the set of marker genes characterizing mouse naïve pluripotency^51^ does not exactly apply to primate pluripotent stem cells^50^. While *REX1* and *FGF4* are expressed in primed human pluripotent stem cells^50^,^52^,^53^, *FGF5* is not upregulated in primed pluripotency^50^. Only little is known about *NROB1* (also known as *DAX1*) in human pluripotent stem cells. However, mouse ES cells with a stable knockdown of *NROB1* were predisposed to differentiation but still maintained pluripotency^54^. T is a marker of differentiating rhesus monkey ESCs^55^,^56^. *REX1* expression was robust in all samples tested, and the average expression was comparable between the novel ESC lines, the previously established ESC line cjes001^7^, our previously generated iPSC line^30^ and neonatal and adult marmoset skin fibroblasts. In contrast, *NROB1* expression was hardly detectable in any of the cell lines except for DPZcjESC4. Moderate *FGF4* expression was detected in all ES cell lines and the iPS cell line but not in the fibroblasts. In contrast to *FGF4*, *FGF5* expression was not detected in any of the novel ES and iPS cell lines, while very weak *FGF5* expression was detected in the established ESC line cjes001 and in neonatal skin fibroblasts. Expression of Brachyury (*T*) was detected at different levels in all cell types except for the marmoset fibroblasts. In summary, quantitative analysis of these marker transcripts indicates a state of primed pluripotency of the novel marmoset monkey ES cells under the conditions used in this study.

## Discussion

We report the derivation of four novel embryonic stem cell lines from natural preimplantation embryos of the common marmoset monkey (*Callithrix jacchus*). Although ESCs are usually derived from the inner cell mass of a blastocyst, it was previously shown that it is also possible to derive ESCs from embryos of earlier stages in mouse and human^57^, and even clonal lines from dissociated, single mouse and human blastomeres could be obtained^58^. However, to the best of our knowledge, all published non-human primate ES cell lines so far were derived from the inner cell mass of a blastocyst^2^,^3^,^5^,^6^,^7^. Here, we report for the first time the derivation of three marmoset ES cell lines from natural morula stage preimplantation embryos. One additional cell line was derived from a blastocyst stage embryo. Prior to culturing, we did not remove the trophoblast by immunosurgery as it is usually done during ES cell line establishment^2^,^3^,^5^,^6^,^7^. Our cells keep their pluripotent state and proliferate in standard embryonic stem cell medium, i.e. without the addition of bFGF or other growth factors.

We could not detect any obvious differences between morula- and blastocyst-derived cell lines – neither in morphology or growth characteristics nor on the molecular level. RT-qPCR did not reveal any statistically significant differences in the expression levels of selected pluripotency genes (data not shown) and also whole transcriptome analyses by deep sequencing proved the absence of global differences between the cell lines of different embryonic origin. In-depth analyses of the transcriptome data revealed differentially expressed genes. However, to prove that potential differences are due to the embryonic origin and not to other parameters, more (euploid) cell lines are needed.

Two of the four novel ES cell lines generated in this study, one male and one female line, have a normal karyotype while karyotype analyses revealed trisomies in the male cell line DPZcjESC1 and in the female cell line DPZcjESC2. According to karyotype analysis, chromosomes 18 and 19 are affected in DPZcjESC1, while global transcriptome data generated by deep sequencing and subsequent transcript mapping indicated trisomies of chromosomes 19 and 20. Analogously, in DPZcjESC2 there are three copies of either chromosome 19 or 20, depending on the method of analysis. Since we were not able to unambiguously bring in line the banded chromosomes 19 and 20 from two different publications^32^,^46^ and since the use of transcriptional profiling is a well-established technique^59^ we conclude that the karyotypes of our cells are [48, XY, +19, +20] for DPZcjESC1 and [47, XX, +20] for DPZcjESC2 as revealed by transcriptome analysis. However, an international agreement on a system for common marmoset monkey cytogenetic nomenclature according to the System for Human Cytogenetic Nomenclature^45^ would be highly desirable to prevent future confusion regarding cytogenetics in the common marmoset monkey.

Irrespective of the karyotypic characteristics of the four lines (that are also reflected in the transcriptomes), we did not observe any obvious differences between the euploid and aneuploid cell lines. Therefore, it still remains to be investigated in detail whether all cell lines have the same characteristics. Moreover, despite the unresolved issue of cytogenetic nomenclature, it would be very interesting to determine which human chromosomes correspond to the marmoset trisomic chromosomes in the novel ES cell lines in order to find out which human trisomies may be (partially) represented by the DPZcjESC lines 1 and 2. However, ignoring the difficulties of chromosome assignment and nomenclature described above, according to Sherlock *et al*.^46^, the entire marmoset chromosomes 18 and 19 show homology with human chromosome 1. This is particularly important because there are only a few autosomal trisomies that are compatible with survival to birth in humans^60^,^61^,^62^,^63^. Previously, it was suggested that the vast majority of human preimplantation embryos resulting from *in vitro* fertilization (IVF) are a mosaic of euploid and aneuploid blastomeres^64^. Tremendous 90% of the analyzed three- and four-day-old human cleavage stage embryos, most of them at the eight cell stage, showed chromosomal abnormalities. It was shown that large-scale structural chromosomal imbalances occurred in 70% of all embryos tested^64^. It is discussed that these high rates of aneuploidy observed in IVF embryos might either be an artefact of *in vitro* manipulation or that early human embryos undergo a unique, transient phase of chromosomal instability^64^. Interestingly, karyotype analysis showed that both of our cell lines obtained from early, non-compacted morulae (DPZcjESC1 and 2) are aneuploid while the remaining two cell lines (DPZcjESC3, DPZcjESC4) derived from later preimplantation stages, are euploid. Importantly, all four lines were obtained from natural, non-manipulated preimplantation embryos of the common marmoset monkey presaging that aneuploidy observed in human IVF embryos may also occur in naturally conceived primate embryos. While it is impossible to study naturally conceived early human embryos and since rodent embryos are not always an appropriate model for human embryology^65^, the marmoset could be a useful primate model species to further study the clinically relevant issue of aneuploidy in primate preimplantation embryos.

Transcriptome analysis of the ES cell lines revealed an evident inverse correlation between *XIST*-expression and transcriptional activity of X chromosomal genes. This allows the conclusion that X-inactivation in genotypically female ES cells of the common marmoset monkey occurs in a *XIST*-dependent manner. However, X inactivation does not seem to be an essential inherent characteristic for pluripotency *in vitro*^66^. Our data suggest that marmoset ES cells, with regard to X-inactivation, in general rather resemble human than mouse ES cells. For undifferentiated mouse ES cells, both X chromosomes are supposed to be active while X-inactivation was reported to be a feature of mouse ES cell differentiation^67^,^68^. In contrast to the mouse, human ES cells, which were reported to be generally in a primed state (in contrast to the mouse ES cells) do not display a consistent pattern of X-inactivation but rather show a high degree of variation between cell lines and even between sub-lines of the same parental cell line reviewed in ref. 69.

Quantitative analysis of the expression of marker genes for naïve or primed pluripotency of the common marmoset monkey ES cells suggests that the novel NHP ES cells are in a primed state of pluripotency (Fig. 7). We attempted to further characterize the state and the plasticity of the cells by culturing both euploid ES cell lines on Matrigel- and Vitronectin-coated plates under primed conditions^70^ and under naïve conditions in 2i-LIF media^71^. Under MEF-free primed conditions, the marmoset ES cells survived maximally for three days and then died (data not shown). Under the 2i-LIF conditions the cells proliferated and kept their colony-like morphology. However, the expression levels of *OCT4A* and *NANOG* dramatically decreased within a few days to less than 2% compared with the levels of cells cultured on MEFs (data not shown). We conclude that culture protocols established for human pluripotent cell are not directly applicable to these marmoset ES cell lines and that adaptations of the protocols for the feeder-free culture of human cells are necessary to enable feeder-free marmoset monkey ES cell culture.

The four novel ES cell lines derived in this study extend the available repertoire of marmoset monkey ES cell lines. Since ES cells derived from natural embryos are considered as the gold standard for pluripotent stem cells, their availability is crucial to identify fundamental (epigenetic) differences between primate ES and induced pluripotent stem (iPS) cells. Furthermore, pluripotency of the novel ESC lines 3 and 4, although apparently in a primed state under culture conditions currently established, may be worth testing in chimera formation assays.

Hierarchical clustering showed that the novel ES cell lines’ transcriptomes are very similar, but that they – together with the transcriptomes of an iPS cell line we previously generated^30^ – separate from the transcriptome of the established ES cell line cjes001. A deeper analysis revealed nearly 5000 differentially expressed genes between these two groups (our ES and iPS cells vs. cjes001 and fibroblasts). Top-20 enriched gene ontology (GO) terms are shown in Fig. S6 (our ES and iPS cells vs. cjes001 and fibroblasts) and S7 (our ES cells vs. cjes001). These data show that, although the culture conditions of the ES cell lines were identical and the cell lines phenotypically indistinguishable, there are clear differences between different cell lines on the transcriptomic level. This emphasizes the substantial need for the availability of an extended set of marmoset monkey ES cell lines for functional and preclinical studies.

Finally, two of our novel cell lines are female and two are male allowing the investigation of possible sex-specific differences between the lines. Interestingly, except for one ES cell line, which unfortunately is not available any more^2^, all ES cell lines from the common marmoset monkey published so far were female^2^,^6^,^7^.

## Conclusion

In the context of the increasing interest in use of the common marmoset as a non-human primate model species that closely resembles human physiology (reviewed in refs 28,29), the four novel marmoset ES cell lines derived in this study are of great value. We previously generated marmoset monkey iPS cells by non-viral means^30^ with the long-term goal to develop and test cell replacement therapies in preclinical settings. Particularly in our ageing societies, cell replacement therapies based on pluripotent stem cells may become a promising option for the treatment of age-associated degenerative diseases. The availability of embryonic and induced pluripotent stem cells, both generated from the same marmoset colony, will allow elaborate comparative translational studies elucidating the potential of each cell type to restore functions of degenerated cells or tissues.

In summary, we have generated four novel ES cell lines from the common marmoset monkey, which exhibit significant differences with regard to their transcriptomes and genomes. The availability of these novel ES cell lines will further promote biomedical research in this NHP species, which is gaining more and more interest in translational studies including cell replacement therapies^21^,^72^.

## Additional Information

**How to cite this article**: Debowski, K. *et al*. The transcriptomes of novel marmoset monkey embryonic stem cell lines reflect distinct genomic features. *Sci. Rep.* **6**, 29122; doi: 10.1038/srep29122 (2016).

## Supplementary Material

Supplementary Information

## Figures and Tables

**Table 1:** Embryonic stem cell lines derived in this study.

**Cell line**	**Embryo donor no**. **and age at embryo retrieval**	**Day post ovulation of embryo retrieval**	**Developmental stage of embryo**	**Culture period of primary outgrowth before first passage**	**Karyotype**
DPZcjESC1	#14637 (3.9 years)	4	Early morula	21 days	48, XY, +18, +19
DPZcjESC2	#14637 (3.9 years)	4	Early morula	21 days	47, XX, +19
DPZcjESC3	#13681 (7.3 years)	6	Compacted morula	15 days	Normal, 46, XX*
DPZcjESC4	#14204 (5.9 years)	5	Expanded blastocyst	17 days	Normal, 46, XY

(*) Individual aberrations were detected in four out of 30 cells.
