# Supplementary material for: The transcriptomes of novel marmoset monkey embryonic stem cell lines reflect distinct genomic features
Source: Sci Rep. 2016 Jul 7;6:29122. doi: 10.1038/srep29122 (PMC4935898; doi:10.1038/srep29122)
Supplement: Supplementary Information [file srep29122-s1.doc]

# Supplemental material

# The transcriptomes of novel marmoset monkey embryonic stem cell lines reflect distinct genomic features

Katharina Debowski, Charis Drummer, Jana Lentes, Maren Cors, Ralf Dressel, Thomas Lingner, Gabriela Salinas-Riester, Sigrid Fuchs, Erika Sasaki, Rüdiger Behr

S1_Tab.: Oligonucleotides used in this study.

| **Fragment** | **Primer name (orientation), primer sequences (5ˈ🡪3ˈ)** | **PCR product (bp)** |
| --- | --- | --- |
| *SOX2* | G0086 (fwd): TCTTCCTCGCACTCCAGGGC | 228 |
|  | G0030 (rev): CCGCTCGAGAATGCCTCCCCCGTCCAGTTCG |  |
| *OCT4A* | G0022 (fwd): GATCGGATCCTTGGGGCGCCTTCCTTC | 510 |
|  | G0035 (rev): CAGGGTGATCCTCTTCTGCTTC |  |
| *KLF4* | G0091 (fwd): GGAAGACGATCTTGGCCCCG | 323 |
|  | G0021 (rev): GTACTCTAGACAGTGTGGGTCATATCCACTG |  |
| *cMYC* | G0079 (fwd): ATAAGAATGCGGCCGCACTGGATTTTTTTCGGGCAGTGG | 456 |
|  | G00142 (rev): CCTGGATGATGATGTTTTTGATG |  |
| *LIN28* | G0305 (fwd): GACGAGCTGTACAAGGGGAGTGAGAGGCGGCCAAAGGGG | 334 |
|  | G0025 (rev): GACTCTCGAGATAGCCAAAGAATAGCCCC |  |
| *NANOG* | G0018 (fwd): GATCAAGCTTCCTTTTCCCCAATAATAACATG | 754 |
|  | G0075 (rev): TTATAGAAGGGACTGCTCCAGG |  |
| *SALL4* | G0104 (fwd): GGCTCGGATAAACGTGGAAGG | 328 |
|  | G0105 (rev): GTTTGCCATGATGGCTTCCTTAG |  |
| *β-ACTIN* | G0336 (fwd): GACGACATGGAGAAGATCTGG | 562 |
|  | G0337 (rev): GGAAAGAAGGCTGGAAGAGTG |  |

S2_Tab.: Antibodies used in this study.

| **Antigen** | **Supplier** | **Order no.** | **Dilution** |
| --- | --- | --- | --- |
| SSEA-4 | Millipore | MAB4304 | 1:50 |
| SSEA-3 | Millipore | MAB4303 | 1:50 |
| SSEA-1 | eBioscience | 14-8813-82 | 1:50 |
| TRA-1-60 | eBioscience | 14-8863 | 1:50 |
| TRA-1-81 | eBioscience | 14-8883 | 1:50 |
| SALL4 | abcam | ab57577 | 1:200 |
| SOX2 | Cell Signaling | 3728 | 1:200 |
| OCT4A | Cell Signaling | 2890S | 1:100 |
| KLF4 | R&D | AF3640 | 1:50 |
| NANOG | Cell Signaling | 4903 | 1:300 |
| LIN28A | Cell Signaling | 3978S | 1:70 |
| CHD1 | BETHYL | A301-218A | 1:200 |
| UTF1 | Millipore | MAB4337 | 1:1000 |
| **Secondary antibodies** | | | |
| Alexa488-dk-α-mm IgG | LifeTech | A21202 | 1:200 |
| Alexa488-dk-α-rb IgG | LifeTech | A21206 | 1:200 |
| Alexa488-dk-α-gt IgG | LifeTech | A11055 | 1:200 |
| Alexa488-dk-α-mm IgG + IgM | LifeTech | A10680 | 1:200 |


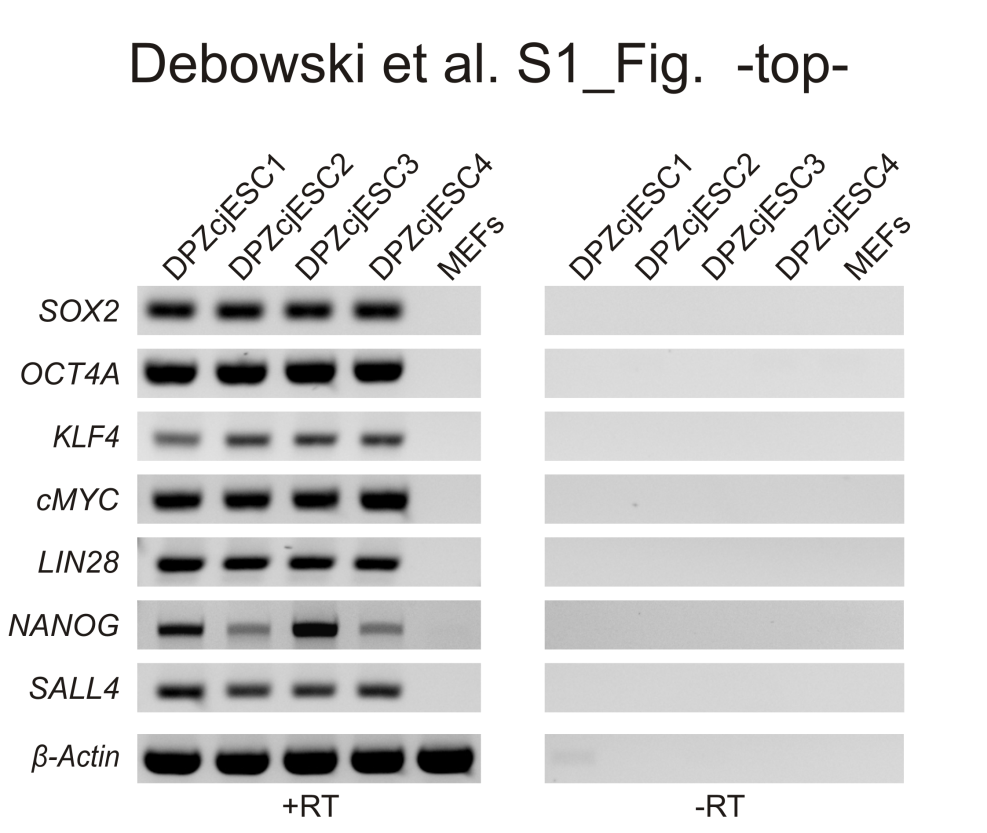
S3_Figure

**S3_Fig.: PCR analysis for pluripotency marker expression.** All generated ESC lines showed expression of mRNA coding for well-established pluripotency-associated factors. Mouse embryonic feeder cells (MEFs) were used as negative control. +RT: PCR analysis with cDNA as template, -RT: negative control PCR where the reverse transcriptase (RT) was omitted during reverse transcription of mRNA.

S4_Figure


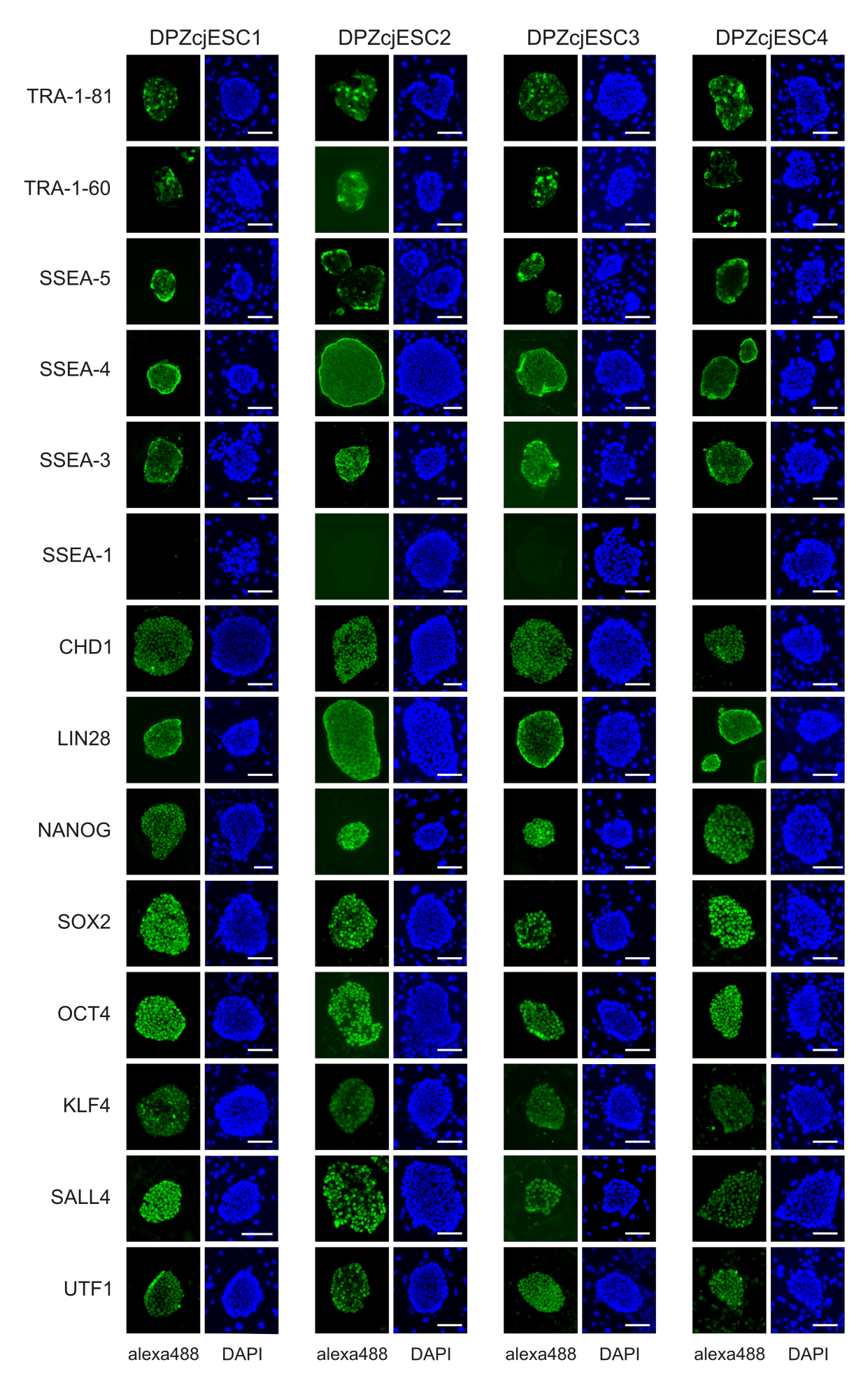


**S4_Fig.: Immunofluorescence staining of ES cell colonies.** Antibodies directed against pluripotency-associated proteins confirmed expression of the surface proteins TRA-1-81, TRA-1-60, SSEA-4 and SSEA-3, the Chromodomain Helicase DNA Binding Protein 1 (CHD1), the RNA binding protein LIN28 and several transcription factors (NANOG, SOX2, OCT4, KLF4, SALL4, UTF1). As expected, SSEA-1 was not detected. All bars = 100 µm.

S5_Figure


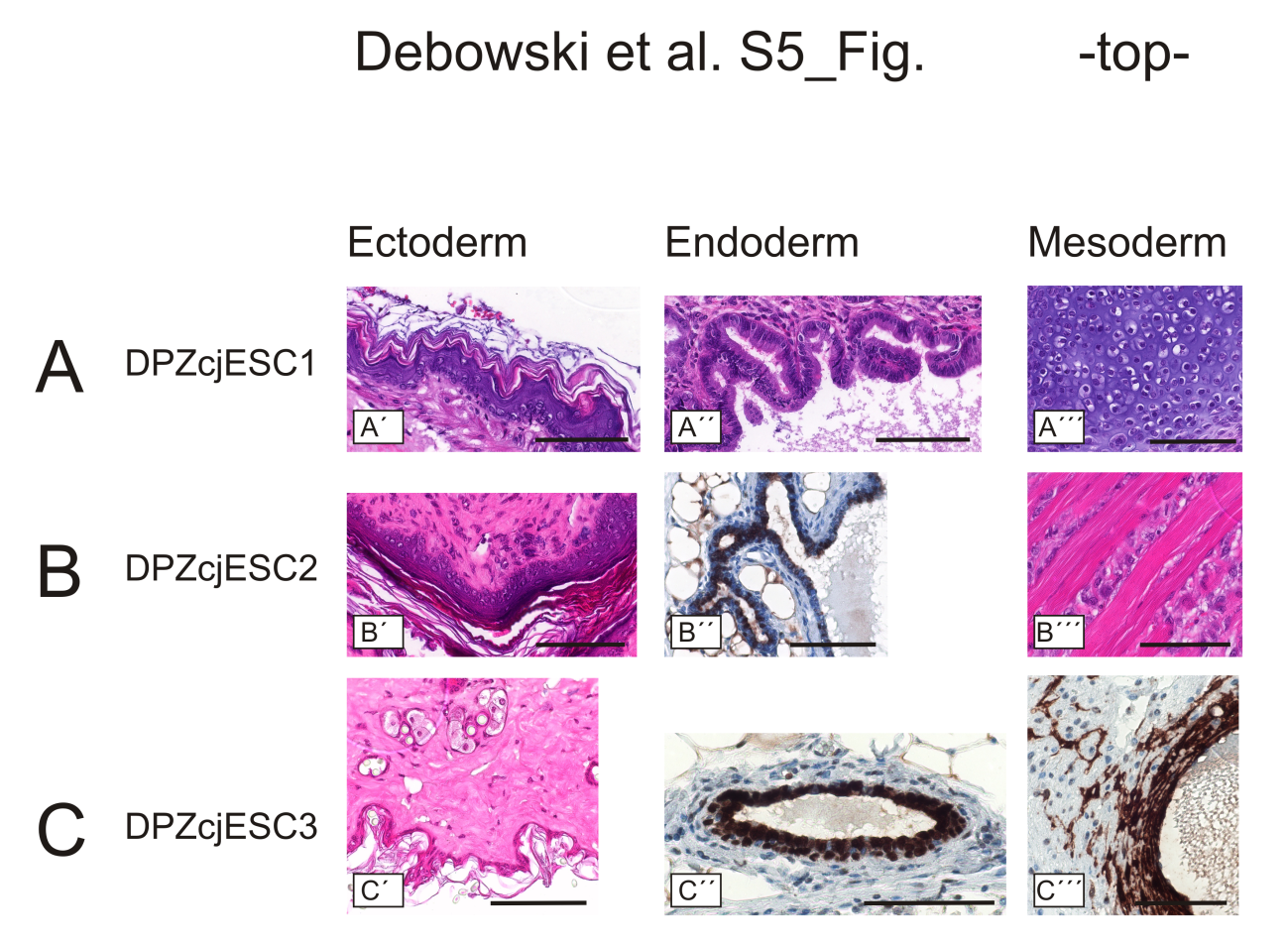


**S5_Fig.: Teratoma assay to test for pluripotency.** HE staining and immunohistochemical analysis of teratoma tissue derived from the cell lines DPZcjESC1-3. Tissues representative of the three embryonic germ layers are shown for each cell line. HE staining of ESC1-derived tissues demonstrates the presence of ectodermal epidermis (**A**A´), endodermal gut-like epithelium (**A**A´´) and of mesodermal cartilage (**A**A´´´). ESC2-derived tissues include ectodermal epidermis (**B**B´), endodermal epithelium positive for SOX9 (**B**B´´) and mesodermal striated muscle cells (**B**B´´´). ESC3-derived tissues include ectodermal epidermis (**C**C´), endodermal duct epithelium positive for SOX9 (**C**C´´) and mesodermal smooth muscle cells positive for α-smooth muscle actin (**C**C´´´).

S6_Figure

**
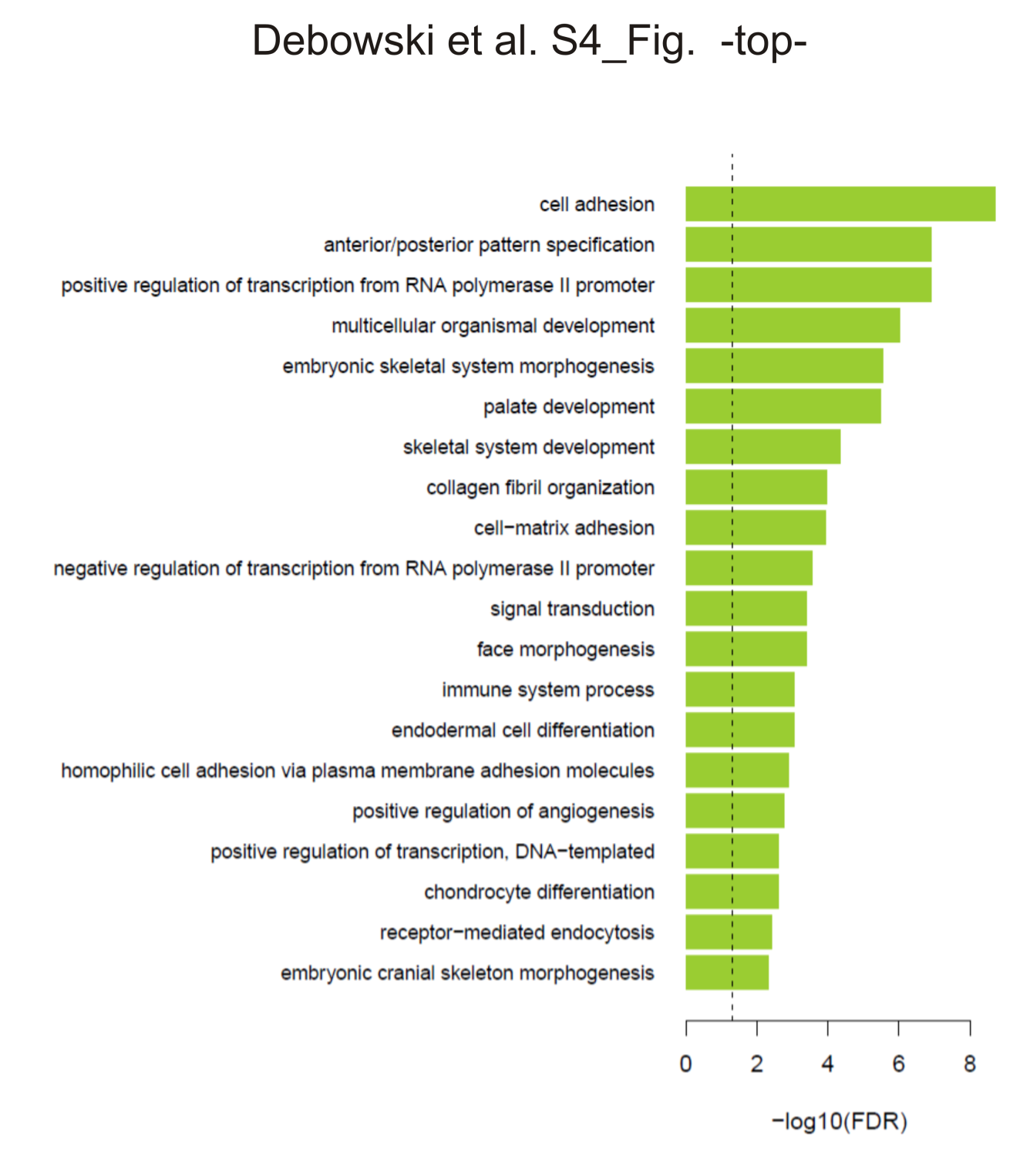
**

**S6_Fig: Gene ontology (GO) analysis.** In order to test the functional association of the candidate genes, an enrichment test for GO terms was conducted for the comparisons DPZcjESC1-4+DPZcj_iPSC1 [^28^](#_ENREF_28)-vs-cjes001+fibroblasts. The top 20 GO terms with the best FDR-corrected p-values were chosen.

S7_Figure


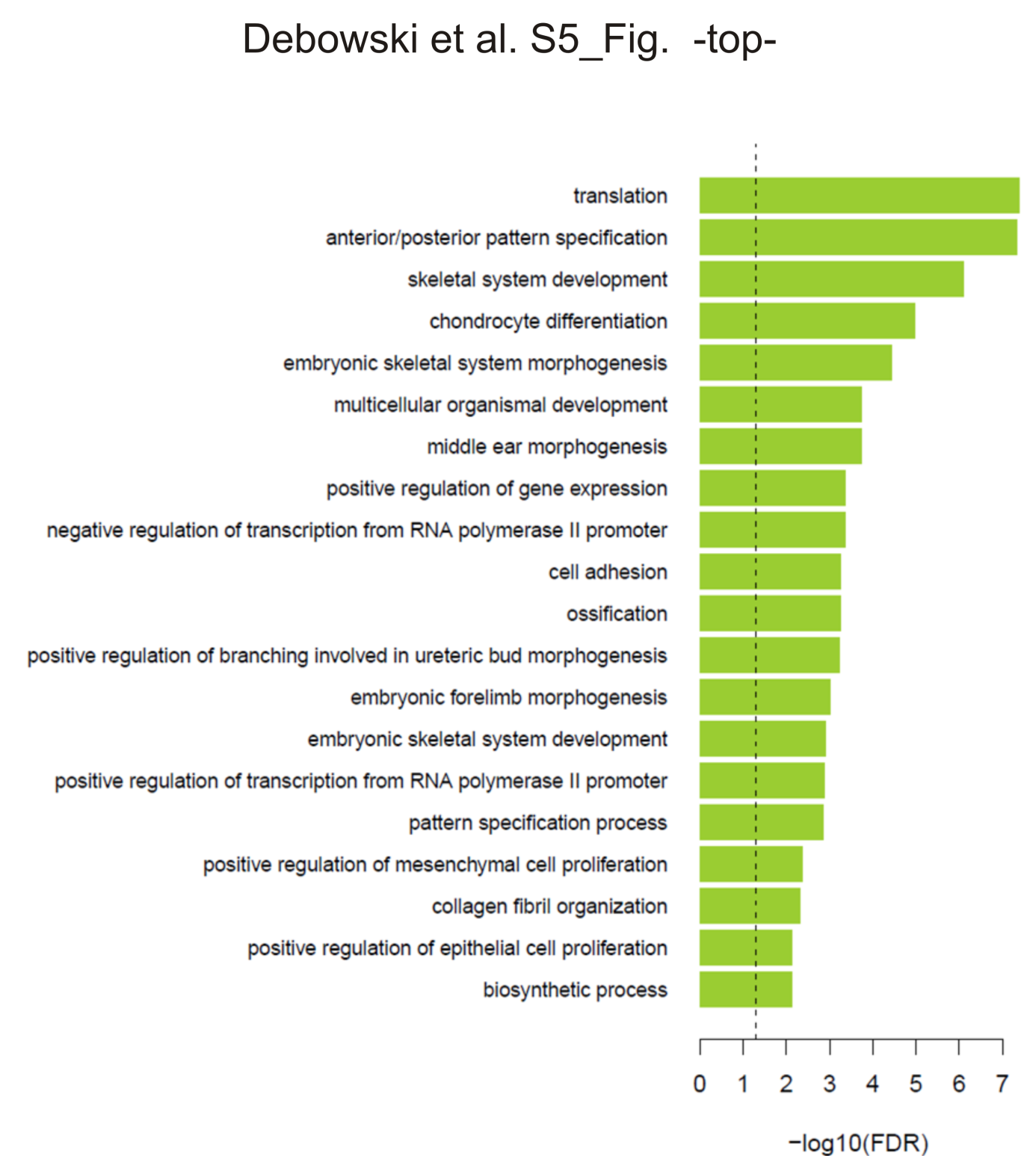


**S7_Fig: Gene ontology (GO) analysis.** In order to test the functional association of the candidate genes, an enrichment test for GO terms was conducted for the comparisons DPZcjESC1-4-vs-cjes001. The top 20 GO terms with the best FDR-corrected p-values were chosen.
